# Phage-encoded carbohydrate-interacting proteins in the human gut

**DOI:** 10.3389/fmicb.2022.1083208

**Published:** 2023-01-06

**Authors:** Daniela Rothschild-Rodriguez, Morgen Hedges, Merve Kaplan, Sercan Karav, Franklin L. Nobrega

**Affiliations:** ^1^School of Biological Sciences, University of Southampton, Southampton, United Kingdom; ^2^Department of Molecular Biology and Genetics, Çanakkale Onsekiz Mart University, Çanakkale, Turkey

**Keywords:** glycosylation, glycans, mucus, mucus-binding, mucus-degrading, mucins, bacteriophage, gut

## Abstract

In the human gastrointestinal tract, the gut mucosa and the bacterial component of the microbiota interact and modulate each other to accomplish a variety of critical functions. These include digestion aid, maintenance of the mucosal barrier, immune regulation, and production of vitamins, hormones, and other metabolites that are important for our health. The mucus lining of the gut is primarily composed of mucins, large glycosylated proteins with glycosylation patterns that vary depending on factors including location in the digestive tract and the local microbial population. Many gut bacteria have evolved to reside within the mucus layer and thus encode mucus-adhering and -degrading proteins. By doing so, they can influence the integrity of the mucus barrier and therefore promote either health maintenance or the onset and progression of some diseases. The viral members of the gut – mostly composed of bacteriophages – have also been shown to have mucus-interacting capabilities, but their mechanisms and effects remain largely unexplored. In this review, we discuss the role of bacteriophages in influencing mucosal integrity, indirectly *via* interactions with other members of the gut microbiota, or directly with the gut mucus *via* phage-encoded carbohydrate-interacting proteins. We additionally discuss how these phage-mucus interactions may influence health and disease states.

## Introduction

The human gastrointestinal tract is composed of a variety of epithelial cells, immune cells and tissues that are covered by a mucus layer. This mucus layer is colonized by a vast amount of bacteria, viruses, fungi and protozoa, which are collectively known as the gut microbiome (Qin et al., 2010; Chin et al., 2020; Camarillo-Guerrero et al., 2021; Chibani et al., 2021). Advances in genomics over the last decade have allowed pivotal research in this field, which has shed light on the importance of the gut microbiota in influencing human health and disease, such that it is often recognised as an organ in itself (O’Hara and Shanahan, 2006; Baquero and Nombela, 2012).

Bacteria are major constituents of the gut microbiota (~10^11^ colony-forming units/g of faeces; Sender et al., 2016) and key players in modulating human health and disease, through the regulation of metabolic, nutritional, physiological, and immunological processes in the human ‘host’ (Cani, 2018; Kho and Lal, 2018; Valdes et al., 2018; Fan and Pedersen, 2020; Sorboni et al., 2022). Viruses are also major constituents of the gut microbiota, with prokaryotic viruses – (bacterio)phages – representing 97.7% of the virome (~10^10^ virus-like particles/g of faeces; Lepage et al., 2008; Hoyles et al., 2014; Lim et al., 2015; Gregory et al., 2020; Liang and Bushman, 2021; Townsend et al., 2021). However, the roles gut phages have in influencing human health and disease have only recently started to be acknowledged, and far fewer of their mechanisms are understood (Shkoporov et al., 2022).

Phages have been shown to encode proteins that bind to the mucus layer (Barr et al., 2013; Almeida et al., 2019; Green et al., 2021), and it is also likely that some phage-encoded enzymes are involved in mucus degradation to facilitate prey encounters, given that phages naturally encode these for accessing the bacterial membrane (Latka et al., 2017). The interaction of phages with the gut mucus is thought to influence human health directly, by affecting the human intestinal cells or mucus, and indirectly, by influencing host communities (e.g., predatory-parasitic dynamics and horizontal gene transfer; Barr et al., 2013; Bichet et al., 2021).

Due to the lack of characterised and profiled phages in the gut, there is little clarity as to which gut phage proteins can mediate these mucus interactions and their downstream effects. Therefore, the study of phage-mediated health modulation is severely limited. In order to increase the understanding of this field, here we describe bacterial-mucus interactions and what is currently known about phage-mucus interactions within the human gut. We additionally discuss how phages may modulate human health, with the example of inflammatory bowel diseases (IBDs).

## The dual interplay between mucus mucins and gut microbiota

The intestinal tract contains a mucosal barrier composed of a single layer of epithelial cells joined by tight junctions and covered by a mucus layer (Vancamelbeke and Vermeire, 2017). The mucosal barrier plays a crucial role in maintaining intestinal immune homeostasis (Johansson et al., 2013; Ouwerkerk et al., 2013; Paone and Cani, 2020). This is achieved by establishing a physical and chemical barrier between the microbiota and the epithelial cell lining, while simultaneously acting as a binding anchor and a nutrient source for many beneficial mucus-residing bacteria (Sicard et al., 2017). More recently, seminal studies by Barr and colleagues have also shown the importance of the mucus layer for phage residence in the gut (Barr et al., 2013, 2015). For these reasons, the mucus lining in the gut is at the core of the tripartite relationship between phages, bacteria and the human ‘host’ (Sutton and Hill, 2019; Townsend et al., 2021; Shkoporov et al., 2022).

The mucus layer consists of 95% water, electrolytes, lipids, and proteins – including 1–5% glycosylated proteins called mucins (Bansil and Turner, 2018). Mucins define the structure and function of the mucus layer. They consist of a protein core with a ‘mucin domain’ characterized by centrally located tandem repeats of proline, threonine and serine residues (PTS regions), which are heavily O-glycosylated at the threonine and serine residues. The carboxy- and amino-terminals of mucins mainly undergo N*-*glycosylation at asparagine residues (Bansil and Turner, 2018; Hansson, 2020). Secreted mucins can also contain C-mannosylation at specific tryptophan residues (Perez-Vilar et al., 2004).

**Figure 1:**
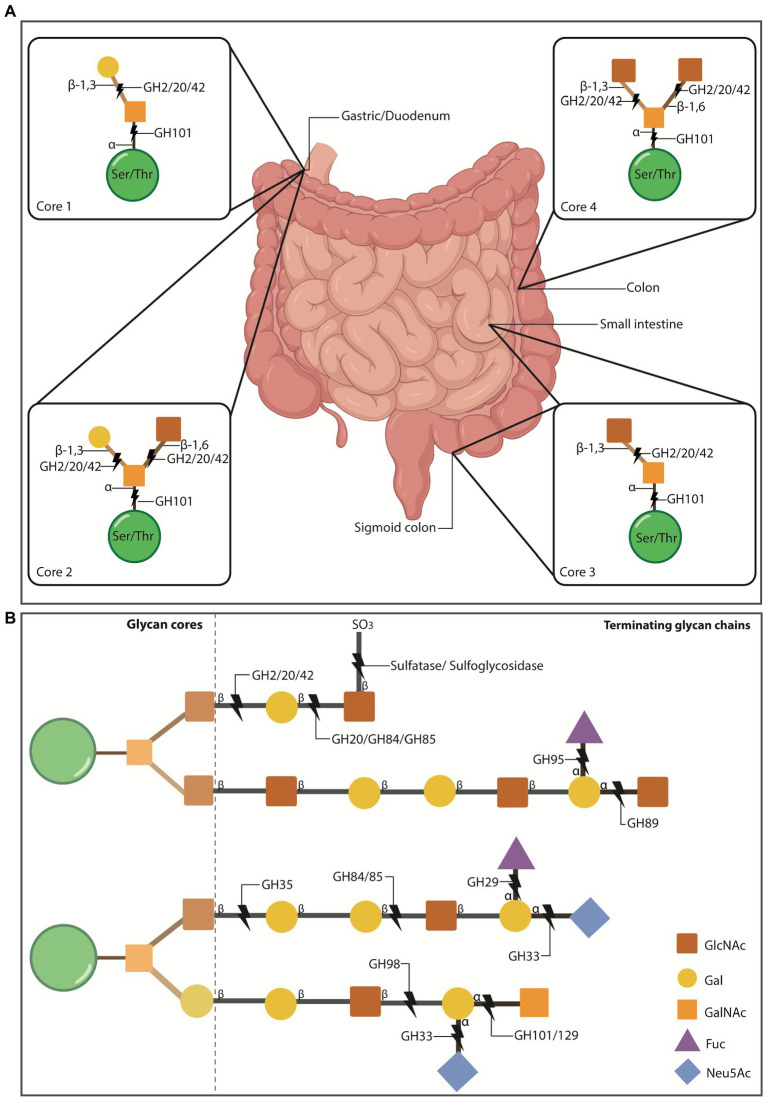
O-glycosylation of mucus mucins in the gut. **(A)** O-glycosylated mucins can contain 1-8 cores that stem off the protein backbone. Cores 1-4 make up the majority of O-glycosylated mucins in the gut. Cores 1 and 2 are found in the stomach and duodenum, core 3 is primarily found in the sigmoid colon and small intestine, and core 4 is localised to the colon. Some mucus-residing bacteria encode glycosyl hydrolases (GH) that cleave mucin glycans for bacteria to use as nutritional sources. This enzymatic breakdown of mucins is also part of the normal mucus turnover. The first N-acetylgalactosamine (GalNAc) of all cores is attached to the ser/thr residues *via* α-1 linkages which can be cleaved by the α-N-acetylgalactosaminidase, GH101. The cores then become differentiated by the addition of more groups to the initial GalNAc. Core 1 has galactose (Gal) attached to GalNAc *via* a β1-3 linkage, which can be cleaved by the β-galactosidases GH2, 20 and 42. Core 2 adds N-acetylglucosamine (GlcNAc) to the structure of core 1 *via* a β1-6 linkage to the GalNAc. The β1-6 and β1-3 bonds can be cleaved by GH2, 20 and 42. Core 3 includes a GlcNAc attached to GalNac *via* a β1-3 linkage, which is susceptible to cleavage by GH2, 20 and 42. Core 4 consists of two GlcNAc molecules bound to the GalNAc *via* a β1-6/β1-3 linkage, susceptible to cleavage by GH2, 20 and 42. Intestinal illustration taken from BioRender.com. **FIGURE 1 (Continued)(B)** Extension example of the glycan chain showing terminating glycans and additional enzymes involved in cleaving these. Gut mucin glycan chains are heavily sulfated (SO_3_), particularly at the distal ends of the intestinal tract, often terminating the chains to protect them from degradation. Sulfatases and sulfoglycosidases are non-GH-enzymes responsible for the desulfation of the chain to allow hydrolysis of the underlying glycans. Released sulfates can be reduced by sulfate-reducing bacteria and thus, their presence also promotes specific bacterial colonisation. Chains can also be terminated with fucose (Fuc) or sialic acid (Neu5Ac) residues that are thought to protect the underlying chain, while also serving as nutritional and energy sources for some bacteria. Neu5Ac is cleaved by sialidases (GH33) and Fuc by fucosidases (GH29 and 95).

The gut is a spatially heterogeneous environment. A major factor contributing to this heterogeneity is the diversity of mucins (>20 encoded in humans) and their differential expression and glycosylation along the gut (Figure 1A; Robbe et al., 2003, 2004), which contributes to the spatial composition and abundance of the gut microbiota (Li et al., 2015; Ringel et al., 2015). Likewise, increasing evidence highlights the essential role the microbiota has in modulating the glycan profile of the mucus layer, and therefore in ensuring its proper function (Wrzosek et al., 2013; Jakobsson et al., 2015; Johansson et al., 2015). Thus, the interaction between the microbiota and mucus mucins is bidirectional.

Moreover, few bacterial species are adapted for the mucosal niche and therefore the density of the microbiota increases towards the distal gut, and towards the lumen (Marcobal et al., 2013; Thursby and Juge, 2017; Glover et al., 2022b). Mucin glycans provide an anchor for those adapted species that reside in the mucosal layer (mucus-adherent bacteria) and serve as nutritional sources for some. *Akkermansia muciniphila*, *Bacteroides* spp., *Bifidobacterium* spp., *Ruminococcus* spp., *Clostridium* spp., *Paraclostridium* spp., and *Prevotella* spp. are all well-known mucin-foragers in the gut (Tailford et al., 2015; Glover et al., 2022b).

Some of these mucus-residing bacteria possess enzymes that allow the degradation of the glycosylation bonds that link mucin glycans, such as glycosyl hydrolases (GH; Glover et al., 2022b), sulfatases (Wright et al., 2000a,b) and sulfoglycosidases (SGL; Rho et al., 2005; Figure 1). Bacteria with such enzymes are known as mucolytic, with *A. muciniphila* and *Bacteroides* spp. being the most studied so far (Glover et al., 2022a). The released oligosaccharides that result after cleavage serve as carbon or nitrogen sources for the effectuating bacteria and surrounding ones (Glover et al., 2022b). *A. muciniphila* for example, has been demonstrated to encode up to nine different families of GHs. Thus, these bacterial-encoded enzymes play a role in mucus turnover as well as in influencing residing microbiota, and ultimately the human ‘host’ (Meijer et al., 2010; Smith et al., 2013; Belkaid and Hand, 2014; Jiao et al., 2020).

Efficient mucin degradation by commensal bacteria relies on synergistic activity from several of these enzymes to break down different linkages that constitute the mucin glycan coat (Figure 1). These enzymes may not come from a single species, but many – and thereby support cross-feeding (Berkhout et al., 2022; Glover et al., 2022a). For instance, mucin glycans terminated with sulfur groups can be targeted by sulfatase and sulfoglycosidase enzymes (Figure 1B), and this initial cleavage can expose remaining glycan linkages to other GH enzymes (Wright et al., 2000a,b; Rho et al., 2005). Terminal sialic acid (Neu5Ac) and fucose (Fuc) residues are cleaved by GH33 sialidases (also known as neuraminidases) and by GH29/95, respectively (Crouch et al., 2020; Figure 1B). The underlying mucin glycan chains can be cleaved by other GH such as N*-*acetylglucosaminidases (GH84, GH85, GH89, GH20), N*-*acetylgalactosaminidases (GH101, GH129) and galactosidases (GH2, GH35, GH42, GH98; Berkhout et al., 2022; Figure 1B). Lastly, large glycan structures can be cleaved by endo-acting O*-*glycanases (GH16). As demonstrated by Glover and colleagues, several gut bacteria can encode multiple copies of GHs that can target internal mucin glycan linkages, supporting the synergistic function of these enzymes in microbiota cross-feeding and homeostasis (Glover et al., 2022a).

Despite their similar abilities in mucus degradation, mucolytic bacteria are differently adapted to the mucus environment. For example, *A. muciniphila,* and *Barnesiella intestinihominis* are known as mucus ‘specialists’, which implies that they solely feed on mucin O-glycans (Derrien et al., 2004; Desai et al., 2016). Most other degraders are considered mucus ‘generalists’ as they can survive from both diet-derived and mucin-derived carbohydrates. In addition, some species have been shown to evolve in response to changes in the nutritional sources available (Desai et al., 2016; Dapa et al., 2022). *Bacteroides thetaiotaomicron* was demonstrated to rapidly evolve and mutate its genome to favour the degradation of mucin-derived glycans in the absence of dietary fibre (Dapa et al., 2022). Similar responses have been seen in *Bacteroides caccae* (Desai et al., 2016). Thus, mucin degradation in the gut is truly a collaborative effort between several bacterial species, which is further influenced by other external factors.

Commensal bacteria in the gut contribute to the normal turnover of the mucus layer, but when colonised by pathogenic bacteria, excessive mucus-degrading activity can lead to pathogenesis and disease states (Nakjang et al., 2012). Such is the case for the enterohemorrhagic *Escherichia coli* (EHEC) that expresses the powerful protease C1 esterase inhibitor, better known as StcE, which strongly degrades mucin glycans (mucinase; Malaker et al., 2019). StcE mediates EHEC pathogenesis by harshly degrading mucins in the mucosal lining of the human gut, exposing the epithelia to the pathogen (Grys et al., 2005). Other pathogens express similar virulent mucinases that contribute to disease states (Sauvaitre et al., 2022).

In addition to mucin-degrading enzymes, bacteria also express mucin-binding proteins and domains. For instance, bacterial pili/fimbriae and flagella have been seen to mediate secondary mucin-binding functions (van Klinken et al., 1995; Derrien et al., 2010). Thus, bacterial glycans have been well-studied, and in recent years, research focused on glycan degradation by bacterial enzymes has given much clarity on their importance in modulating human health (Thompson et al., 2017; Fan and Pedersen, 2020; Alemao et al., 2021).

**Figure 2:**
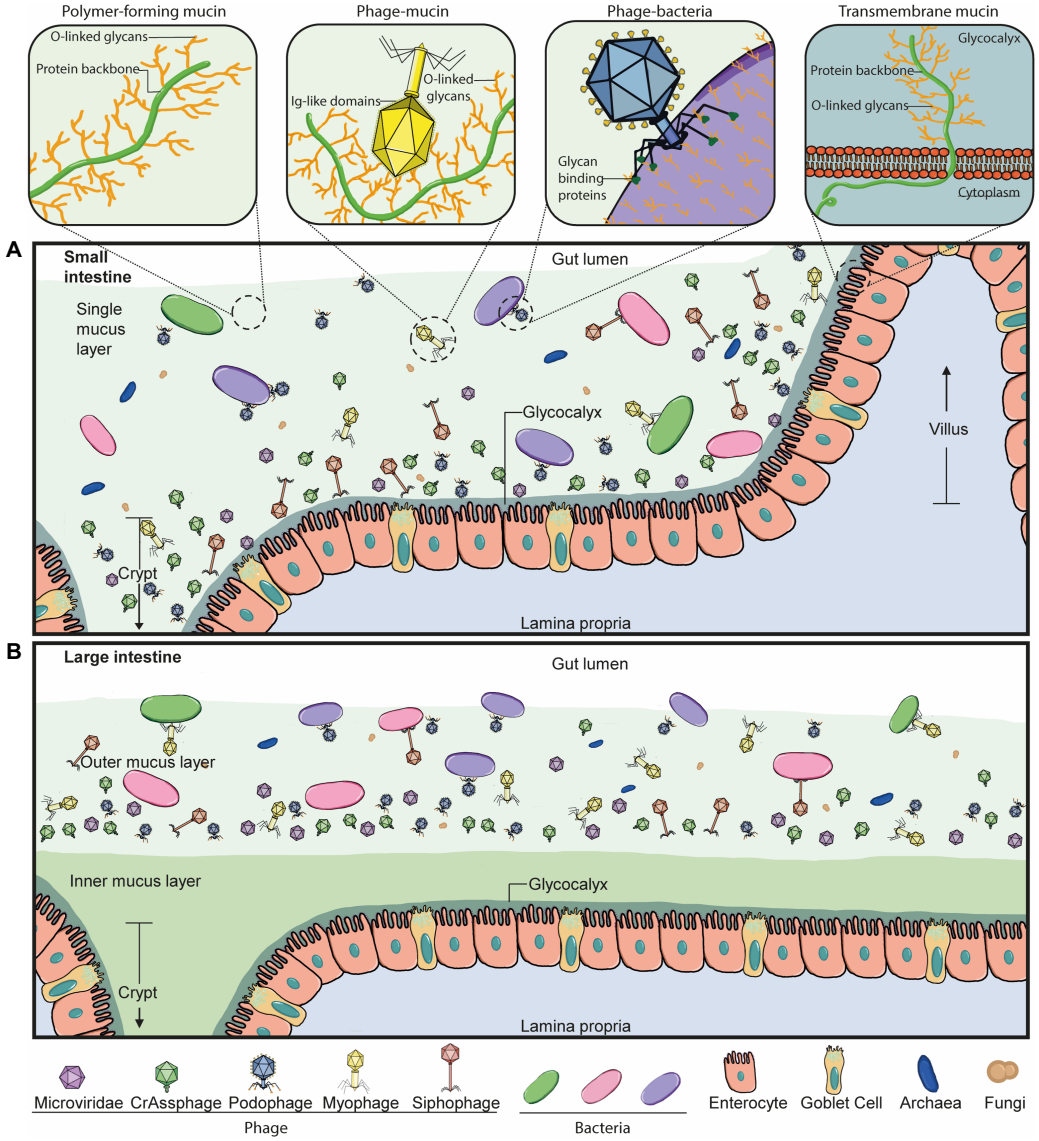
Phage-mucus interactions in the human gut. **(A)** In the small intestine, the microbiota can penetrate and reside in the single mucus layer, which is produced by goblet cells found within the epithelium layer. Phages can interact with and anchor to intestinal mucus in a suggested diffused gradient towards the lumen. This interaction allows the phage and host to be in close contact and co-exist. The mucus structure and function are defined by mucins, which are glycosylated with a high percentage of O-linked glycans. Some double-stranded DNA phages bind to mucus mucins *via* immunoglobulin-like (Ig-like) domains of the highly immunogenic outer capsid (Hoc) protein. Phage tail fibre proteins recognise glycans on the bacterial cell surface and thereby mediate phage adsorption to their host to initiate the infection process. Transmembrane mucins span the enterocyte lipid bilayer to form the glycocalyx. They consist of a cytoplasmic tail, protein backbone and mostly O-linked glycans. **(B)** The large intestine is comprised of an outer and inner mucus layer, but only the former is penetrable to the microbiota. Phages are suggested to co-exist with their hosts in this outer mucus layer in a similar diffused gradient to the small intestine. The dense, inner mucus layer of the large intestine consists of a tighter mesh of mucins with long branches of O-glycans that prevent bacteria-gut epithelium interactions in a healthy context.

On the other hand, mucus-interacting properties by other members of the microbiota are less well understood. We know phages also inhabit the mucus layer in the gut in a spatially heterogeneous manner (Figure 2; Barr et al., 2015; Lourenço et al., 2020). In fact, phage populations are thought to increase by 4-fold in the mucus layer compared to the surrounding environment (Barr et al., 2013). Phages are also known to encode several carbohydrate-binding and -degrading proteins to target their natural hosts, bacteria and archaea (Figure 2A, Phage-bacteria), which have been strongly suggested to also mediate their binding to mucosal surfaces, including mucus mucins (Figure 2A, Phage-mucin) – a hypothesis now demonstrated for several phages (Barr et al., 2013; Almeida et al., 2019; Green et al., 2021).

## Interaction of phages with the human ‘host’

As natural residents of the human gut microbiome, phages interact with the human ‘host’ directly and/or indirectly through their bacterial prey. Indirect interactions have been extensively reviewed by others (see Shkoporov and Hill, 2019; Guerin and Hill, 2020; Shkoporov et al., 2022), and thus we will discuss direct phage-human interactions. These include recognition and/or binding and phagocytosis by components of the immune system, epithelial cell interactions and transcytosis, as well as mucus interactions.

Firstly, the immune system can interact with phages by phagocytosis (Hodyra-Stefaniak et al., 2015) and by the production of antibodies against these, including IgM, IgG and IgA (Gembara and Dąbrowska, 2021). Cytokine production and complement system activation has also been seen in response to phages (Park et al., 2014; Hodyra-Stefaniak et al., 2015) with conflicting results (van Belleghem et al., 2018), which may be explained by variability between phages. For instance, some phages may be pro-inflammatory while others initiate anti-inflammatory responses (Federici et al., 2022a,b).

Within mucosal tissues, IgA is the predominant antibody isotype, produced daily in high amounts (Woof and Ken, 2006). The roles of phage-IgA interaction in modulating human health have not been investigated. However, binding of IgA to some gut bacteria, in addition to immune regulatory roles (Peterson et al., 2007), has also been suggested to be important for successful colonisation of some mucosal-dwelling bacteria, and for maintenance of the bacterial metabolic rhythmicity (Kubinak and Round, 2016; Sutherland et al., 2016; Donaldson et al., 2018; Rollenske et al., 2021; Penny et al., 2022). Thus, we suspect similar roles are relevant in phages but studies are warranted to underpin the roles and mechanisms involved. It will be important to consider the mechanisms of phage-antibody binding when trying to understand these interactions (e.g., cross-specific and polyspecific binding, canonical and non-canonical binding; Pabst and Slack, 2019).

Phage-epithelial interactions were first demonstrated using the filamentous M13 phage as vectors (phage display library), where these vector phages were internalised by the mucosal epithelia (Duerr et al., 2004; Costantini et al., 2009). Subsequently, several other phage types were shown to transcytose through the epithelia using *in vitro* models, *via* non-specific mechanisms (Nguyen et al., 2017). These mechanisms and their effects on the human ‘host’ also warrant further investigation.

Lastly, phage-mucus interactions in the gut were first demonstrated in the seminal work by Barr et al. (2013). This work triggered the start of this new field of research studying phage-mucus interactions, which the remaining part of this review will focus on.

## Interaction of phages with glycans

### Glycan-binding

**Figure 3:**
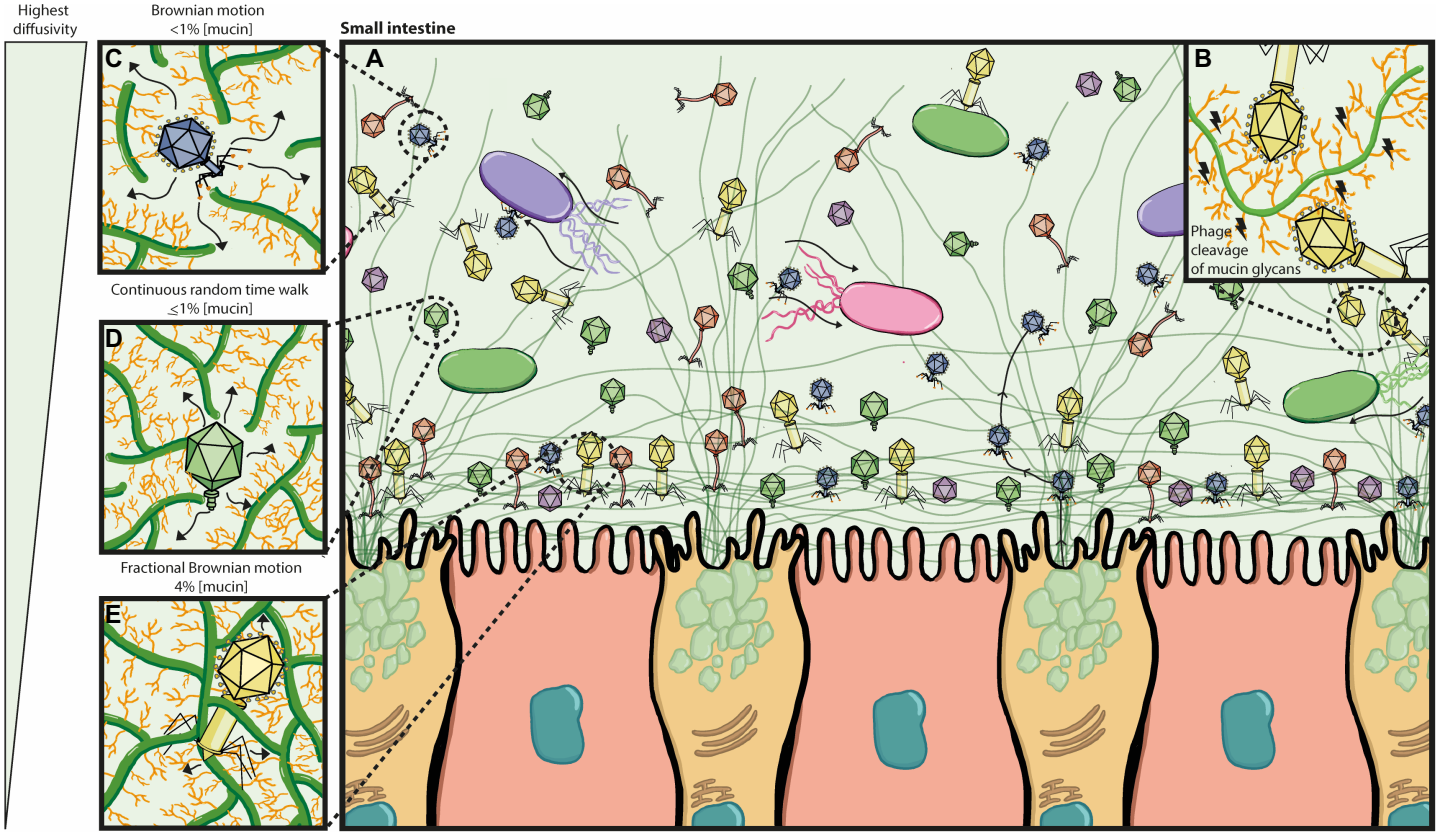
Bacteriophage adherence to mucus (BAM) model expanded. **(A)** The mucus layer exists as a dynamic gradient with a higher density of glycoproteins closer to the epithelia, which is thought to cause a phage gradient within the mucus layer. The phage gradient consists of higher concentrations of phage closer to the epithelia, such that only continuous mucus secretion by goblet cells will push phages back out towards the lumen. Phage residence within the mucus layer allows them to be in close contact with their hosts as bacterial motility through the mucus increases their encounter rates; thus phage-bacterial interactions take place herein. **(B)** Potential phage-encoded carbohydrate active enzymes cleave mucin glycans allowing phages to diffuse down the mucus layer with more ease. **(C–E)** The phage gradient across the mucus layer is attributed to mucus density and phage diffusivity, with highest phage diffusivity occurring at lower mucin concentrations. It is also thought that different phages may have proteins with different binding and/or cleaving affinities to mucin glycans, affecting thus how deeply they can diffuse. **(C)** At <1% mucin concentration, Brownian motion is the dominating diffusion mode (high efficiency). **(D)** At ≥1% mucin concentration, the sub-diffusive continuous random time walk mode predominates (medium efficiency). **(E)** At 4% mucin concentration, the sub-diffusive fractional Brownian motion mode predominates (least efficient). Eventually phages will get stuck on the dense mucin mesh and only pushed out by continuous mucus secretion.

Early work by Fraser and colleagues showed that Ig-like domains are commonly found in tailed double-stranded DNA phages and most often displayed on surface proteins (Fraser et al., 2006). Subsequently, Minot and colleagues identified that human gut phages were rich in hypervariable regions, of which several were predicted to encode C-type lectin folds and Ig-superfamily beta-sandwich domains (Minot et al., 2012), known for mediating carbohydrate-binding interactions (Zelensky and Gready, 2005; Fraser et al., 2006). The authors suggest these hypervariable regions most likely contribute to phage-ligand binding, particularly if they are found on the phage capsid or tail fibres, such as the highly immunogenic outer capsid (Hoc) protein of the coliphage T4 and T4-like phages (Minot et al., 2012). This led to the work by Barr and colleagues that demonstrated that T4 can bind to mucus mucins *via* the Ig-like domains that constitute the hoc protein on the phage capsid. The authors proposed the BAM (bacteriophage adherence to mucus) model (Figure 3) that contributes to a non-host derived immunity against bacterial invasion on mucosal surfaces (Barr et al., 2013). Additional work by the same group provided evidence that the BAM model depends on phage sub-diffusion through the mucosal layer to allow increased encounters with their host (Barr et al., 2015). This increased encounter rate was later suggested to be majorly influenced by bacteria’s motility in an *in vitro* model using the T4 – *E. coli* pair (Joiner et al., 2019).

These works were followed by additional studies investigating phage-mucus interactions in different conditions including rainbow trout, mice, and *in vitro* settings (Almeida et al., 2019; Green et al., 2021; Chin et al., 2022). Almeida and colleagues demonstrated that four of their five tested phages, as well as T4, were able to bind to mucus *in vitro* (FCL-2, V46, PRD1, and FL-1). Phages FCL-2 and T4 were also proven to bind to the skin mucosa of rainbow trout, and FCL-2 binding delayed disease onset by *Flavobacterium columnare*, thereby increasing the survival of the fish. Like T4, the authors confirmed FCL-2 and V46 also contained Ig-like domains and suggested these mediate the binding interactions to mucus. However, the proteins containing these Ig-folds were not tested experimentally, and no Ig-like domains were found in phages PRD1 nor FL-1. Therefore, how these phages bind mucus remains speculative. Green and colleagues identified an enhanced lytic activity of phage ES17 against the extraintestinal pathogenic *E. coli* when the phage is bound to heparan sulfate residues on mucus mucins, *via* the tail fibre protein. Contrastingly, in this same study, the activity of phage HP3 was hampered by the presence of mucins (Green et al., 2021). This suggests that like bacteria, some phages are better adapted to mucus environments than others. In addition to this, phages have also been demonstrated to be able to adapt and evolve in response to their mammalian ‘host’, as well as their bacterial host, thereby allowing their persistence in the mucus layer (Chin et al., 2022).

In unison, these studies have identified specific phages that mediate mucosal binding and thereby provide the phage and/or their host with evolutionary advantages. In some instances, the proteins in the phage mediating the binding have also been uncovered. In addition to these experimental settings that have looked for specific phage-mucus binding, other indirect experimental studies have been previously done that identify phage proteins that may mediate phage-mucus binding, potentially as a secondary function.

For instance, Dong and colleagues identified that the major coat protein (p8) of the coliphage M13, strongly binds to elemental sulfur, in the context of a bioengineering approach (increasing the discharge capacity of lithium batteries by the self-assembly of nanostructured materials; in this case, phage; Dong et al., 2013). Given that some glycan residues in mucus mucins are sulfated (Robbe et al., 2003; Luis et al., 2021), it was proposed by Carroll-Portillo and Lin that M13 could potentially have mucus mucin-binding properties (Carroll-Portillo and Lin, 2021). Similarly, phage P35 has been shown to bind to the N-acetyl group in GlcNAc residues on the cell wall of *Listeria monocytogenes*, *via* the carbohydrate-binding domain P35 (CBDP35; Eugster et al., 2011). Given that GlcNAc is a common residue in O-glycosylated mucus mucins, it has been suggested that CBDP35 could also recognise it in this context (Carroll-Portillo and Lin, 2021). However, it is important to note that this binding is dependent on the structural setting where GlcNAc is found, such that this phage may not necessarily be able to bind to GlcNAc residues in gut mucus mucins (Eugster et al., 2011). In the same paper, Carroll-Portillo and Lin also suggest phage phi187 could have mucin-specificity given that the phage recognised GlcNAc on its host cell wall (*via* unknown mechanisms; Winstel et al., 2014; Carroll-Portillo and Lin, 2021).

Indirect experimental evidence has also suggested mucin-binding properties in phage K5. Green and colleagues found that the tail fibre protein of the mucin-binding phage ES17 (mentioned above), shares high structural similarity to the K5 lyase binding domain (Green et al., 2021). K5 uses its tail spike lyase (KflA) to cleave the GlcNAc and glucuronic acid residues on its host capsule (heparosan), which is identical to the precursor of heparin and hepararan sulfate (O’Leary et al., 2013). The structural similarity between mucins and heparan sulfate led to the suggestion that these phages may be able to target mucins *via* their tails (Green et al., 2021). This highlights the potential of using protein structural resolution or predictions to aid the discovery of more phage-encoded, mucin-interacting proteins.

On this note, indirect experimental evidence of mucus-binding phages also includes crystal structure resolution of some phage proteins. For instance, phage phi11 recognises GlcNAc residues on the host’s cell wall, *via* Gp45, regardless of GlcNAc’s anomeric configurations (Koç et al., 2016; Li et al., 2016). Thus, GlcNAc recognition *via* Gp45 has been extended to mucus mucins (Carroll-Portillo and Lin, 2021). Similarly, the endosialidase (endoNF) on the tail of phage K1F was shown to bind and cleave sialic acid (Stummeyer et al., 2004), and was therefore suggested that phages in the gut may extend these functions to mucin glycans (Carroll-Portillo and Lin, 2021).

Other phages include phi1.2 and phi92, whose substrates have been experimentally tested, albeit not directly with mucin-relevant structures (Kwiatkowski et al., 1983). Both phages encode endosialidases that were shown to cleave sialic acid residues on bacterial capsules, and thus suggested to be relevant in mucin glycan specificity (Carroll-Portillo and Lin, 2021). Only phage phi92, however has had further experimental validation and crystal structure resolution (Schwarzer et al., 2012, 2015). Interestingly, phi92 endoN92 was highly comparable to the mentioned endoNF of phage K1F, although they prefer different linkages: α2,9- and α2,8-, respectively (endoN92 can bind both; Schwarzer et al., 2015). Sialic acid residues in mucin glycans can present α2,8- linkages (amongst others; Cohen and Varki, 2010), suggesting that these enzymes may extend their activity to mucus mucins, subject to experimental validation. Overall, the comparison of protein structures may have potential in uncovering additional mucin-interacting proteins and could be applied before undertaking laborious experimental validation of phage-mucus specificity.

Other computational efforts can also provide valuable information about phage-binding proteins despite the need to corroborate findings experimentally. For instance, analysis of metagenomic data led to the identification of a new carbohydrate-binding module initially found in gut bacteria: the BACON (Bacteroidetes-Associated Carbohydrate-binding Often N-terminal) domain, which was predicted to mediate bacterial binding to mucin glycoproteins (Mello et al., 2010). Subsequently, the BACON domain has been identified in several gut phage sequences, with suggestion of the same function in phage-mucus binding (Reyes et al., 2013; Dutilh et al., 2014; de Jonge et al., 2019). Phage ϕHSC01, identified in mice faeces and originally from virus-like particles (VLPs) purified from human faecal samples, was shown to encode this domain, suggestive of mucus-binding properties (Reyes et al., 2013). Importantly, the discovery of the crAssphage in 2014 not only demonstrated the wide-spread abundance of this phage in the human gut (representative of up to 90% of the reads from VLP-derived gut metagenomes, and found in ~50% of individuals), but additionally identified several BACON domains encoded in the consensus genome sequence of the crAssphage (Dutilh et al., 2014). In addition to the prototypical crAssphage identified *in silico* in 2014 by Dutilh and colleagues, several related crAss-like phages were later identified from metagenomic reads (Guerin et al., 2018; Edwards et al., 2019; Yutin et al., 2021). The discovery of the vast phylogeny of crAss-like phages led to the proposal of organising this taxon into four candidate subfamilies composed of ten candidate genera (Guerin et al., 2018). Current ICTV (International Committee on Taxonomy of Viruses) updates have grouped the crAssphage and crAss-like phages under the *Crassvirales* order with four family types and 11 subfamilies (Walker et al., 2022). A few crAss-like phages have been isolated, confirming *Bacteroides* spp. as their predicted hosts, as well as their ability to co-replicate with their host, possibly contributing to the steady maintenance of crAss-like phages in the gut (Shkoporov et al., 2018; Guerin et al., 2021). Their ability to interact with mucus has also been suggested to play a role in the long-term persistence of these phages in the gut (Guerin et al., 2021; Shkoporov et al., 2021). However, experimental validation is needed to confirm crAss-like phage-mucus interactions.

Moreover, de Jonge and colleagues created Hidden-Markov Models (HMM) to identify BACON domains in phages derived from human gut or sewage datasets (de Jonge et al., 2019). In this computational paper, the authors identified over 1,200 BACON domains among seven lineages of gut phages, including in taxonomically diverse crAss-like phages. Of these seven lineages, only three were predicted to encode the BACON domain in tandem repeats and to be located near tail fibre proteins. One of these three lineages includes the prototypical crAssphage. The authors here suggest phages in these three lineages may have acquired the BACON domain to facilitate interactions with *Bacteroides* spp. Importantly, not all BACON domains may mediate mucin binding (Mello et al., 2010; or bacteria-binding), as experimentally confirmed for the BACON domain present in the endo-xyloglucanase, BoGH5a, which was unable to bind any of the substrates tested (Larsbrink et al., 2014). This finding highlights the importance of corroborating computational data in experimental settings.

Altogether, these studies reveal several phage-encoded mucin-binding proteins (either suggested or demonstrated), although not always specific to the human gut, and importantly, most often demonstrated in *in vitro* settings. Due to the difficulties and challenges of *in vivo* studies particularly in a mucus context, phage-mucus interactions, mechanisms, and potential downstream effects on the human ‘host’, remain unclear and speculative. Additionally, these interactions have mainly been tested between one single pair of phage-bacterium. Almeida and colleagues reported that with the addition of a non-specific phage (T4), the dynamics between the *F. columnare* phage FCL-2 and the host changed (Almeida et al., 2019). Therefore, we need to explore these dynamics and their influence on the human ‘host’ at a population level.

### Glycan-degrading

Phages naturally encode glycan-degrading enzymes, whose primary function is to target the cell wall of their hosts (e.g., bacterial polysaccharides) to allow adsorption and infection (Brüssow et al., 2004; Latka et al., 2017). These enzymes include depolymerases, lysins, and endolysins (Simpson et al., 2015; Latka et al., 2017).

Depolymerases are differentiated into two groups: hydrolases and lyases. These can be further classified on their enzymatic activity and substrate type. Generally, depolymerases contain three domains whereby the central domain mediates receptor binding and catalytic activity (Latka et al., 2017). On the other hand, lysins can be classified as glycosidases (lysozymes, glucosaminidases, and lytic transglycosylases), amidases, and endopeptidases; often found attached to the phage tail. Generally, lysins have a single catalytic domain, sometimes two (Latka et al., 2017). Both depolymerases and lysins are used by the phage during the initial stages of infection. Endolysins are hydrolases often used by the phage at the end of their replication cycle to extensively degrade the peptidoglycan and release the newly formed virions (Schmelcher et al., 2012). These often consist of a cell wall binding domain (CBD) and an enzymatically active domain (Schmelcher et al., 2012).

While a plethora of studies has investigated the activity and mechanisms of these phage enzymes against bacterial polysaccharides, to our knowledge none have focused on their potential in catalytic cleavage of similar residues found on gut mucus mucins. Some of these phage enzymes have been mentioned in the previous section (endoNF, endoN92, HK620TSP), but more reflecting their *binding* potential as no knowledge on their *catalytic* potential against mucins is available.

It is possible that some of these enzymes may have a dual function in targeting mucins of mucosal surfaces that have consequently allowed the evolutionary persistence of phages in mucosal surfaces. One mechanism that supports this hypothesis would be that relevant endolysins bind to mucin glycans *via* the CBD, to allow enzymatic activity (i.e., mucin breakdown) by the catalytic domain. Previous work on BACON domains in gut bacteria has shown that in *A. muciniphila*, for example, the BACON domain is present together with a GH18 domain or a presumed carbohydrate-binding PA14 and a catalytic sulfatase domain, suggestive of binding followed by a catalytic activity that may be beneficial for the bacterium (Mello et al., 2010). Thus, enzymes attached to the phage structures could mediate similar secondary functions. Experimental studies to investigate this hypothesis are lacking and thus warranted.

## Potential effects of phage-mucus interactions on human health

Investigating phage-encoded mucin-interacting proteins will provide a better characterisation of gut phages, and additionally allow for an improved understanding of phage-mediated health modulation and downstream biotechnical and clinical applications. This section briefly summarises what is known about mucosal dysbiosis in disease, with a focus on IBD, and speculates on potential phage-mediated mechanisms that could be playing a role.

Mucosal disruption leads to microbiota dysbiosis, gut inflammation, and impaired epithelial cell function, amongst other negative effects (Choi et al., 2017). This can often lead to the rise or worsening of pathological conditions such as irritable bowel syndrome, IBD, diabetes, obesity, autoimmune disorders and colorectal cancer (Gitter et al., 2001; Mankertz et al., 2007; Groschwitz et al., 2009; Martini et al., 2017; Kinashi and Hase, 2021).

A disrupted mucus barrier and gut inflammation is strongly evidenced in the development of metabolic disorders such as obesity and diabetes, and microbial dysbiosis is suggested to play a role in these effects (Cani et al., 2007, 2008; Wang et al., 2012; Boutagy et al., 2016; Dey et al., 2019; Schroeder et al., 2020). Associations between the virome and these diseases have been more recently established. For instance, an increased abundance of phages, independent of their bacterial hosts, has been demonstrated in patients with type 2 diabetes compared to healthy controls (Ma et al., 2018). Moreover, a study on mice demonstrated that a faecal virome transplantation from lean mouse donors to diet-induced obese mice promoted weight loss, improved glucose tolerance, and induced changes in the metabolome and gene expression similar to those of the lean mice (Rasmussen et al., 2020). Yang and colleagues characterised the virome in obese patients with or without type 2 diabetes and found a decreased viral richness as well as enriched viral species (including *Escherichia*, *Geobacillus* and *Lactobacillus* phages) in obese subjects (Yang et al., 2021). Notably, patients with diabetes have a different virome profile to those without (Yang et al., 2021). Contrastingly, in a separate study, phage richness and diversity increased in children with obesity (Bikel et al., 2021). Overall, the role of phages in the development and pathogenesis of metabolic diseases is unknown due to the lack of studies investigating the mechanisms behind the mentioned viral alterations, calling for more research on this field.

**Figure 4:**
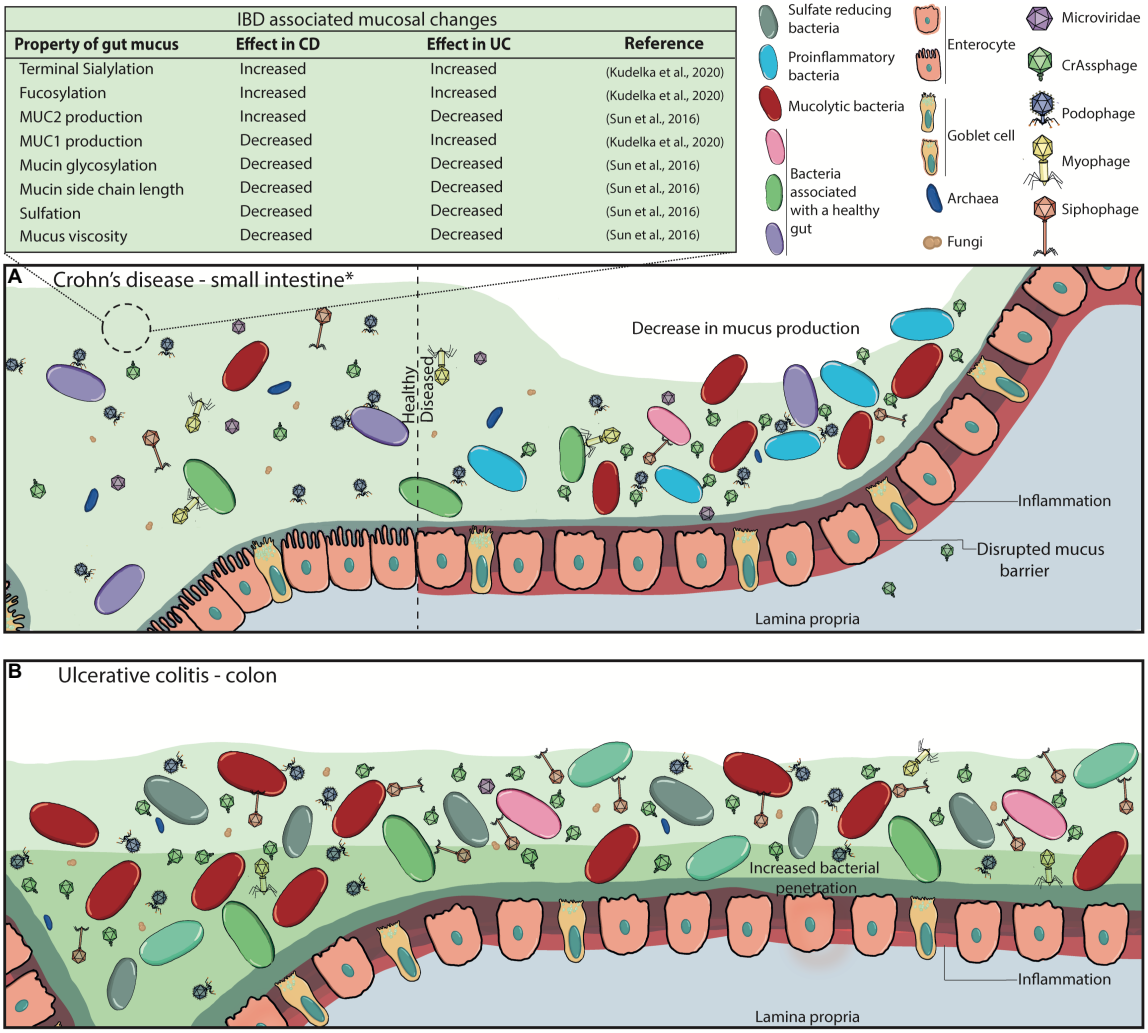
Disrupted mucus barrier and microbiome in Inflammatory Bowel Diseases. **(A)** At diagnosis, Crohn’s disease is most commonly located in the terminal ileum (45%), the colon (32%), ileocolonic (19%), and the upper gastrointestinal tract (4%; *). CD classically presents with discontinuous inflammation (skip lesions) of the gastrointestinal tract, where affected areas will see decreases in mucus production, loss of functional goblet cells, and increased inflammation whilst other sections appear healthy. Commensal bacterial richness and diversity are reduced while pathogenic bacteria and virulent dsDNA phage richness are increased. The mucosal barrier becomes weakened and ‘leaky’, consequently triggering inflammation, and can also lead to phage transcytosis into the lamina propria. **(B)** Inflammation in ulcerative colitis is localised to the colon, and it can continuously affect the large bowel at varying degrees. Virulent dsDNA phage richness, as well as sulfate-reducing, mucolytic and pro-inflammatory bacteria increase, while overall bacterial diversity and richness decrease. Mucus production, secretion and viscosity are substantially decreased, particularly in active disease, leading to the thinning of the mucus layer. This is attributed to the decreased presence of goblet cells in the epithelia, and the increased presence of immature goblet cells that secrete less and faulty mucus with decreased mucin glycosylation. It can also be partly attributed to the increased enzymatic activity of mucolytic bacteria. Eventually, the microbiota can penetrate the inner mucus layer, and worsen inflammatory responses.

In IBD (mainly comprising Crohn’s disease (CD) and ulcerative colitis (UC)), the structure and function of the mucosal barrier is altered (Sun et al., 2016; Grondin et al., 2020; Kudelka et al., 2020). These alterations include differences in mucin expression, glycosylation patterns, and mucus thickness in CD, amongst others highlighted in Figure 4A (Sheng et al., 2012; Sun et al., 2016; Kudelka et al., 2020; Nyström et al., 2021). It has been suggested that these changes lead to the evidenced microbial dysbiosis (including phageome dysbiosis) and downstream immune-associated effects (Figure 4; Mukhopadhya et al., 2012; Sinha et al., 2022; Federici et al., 2022a,b).

The role the microbiota play in IBD-associated mucosal changes (or *vice-versa*) is not well understood. Seminal work by Norman and colleagues provided initial concrete evidence that the enteric virome was altered in patients with IBD, showing an increase in phage richness (Norman et al., 2015). While 85% of the sequences obtained in this study were not matched to any viral taxa, those that fell under *Caudovirales* (currently classified as *Caudoviricetes*) were significantly enriched in IBD when compared to household controls. The authors also demonstrated that the enrichment of phage species within the *Caudovirales* taxa were uniquely distinct between CD and UC (and between the three cohorts studied), with only CD patients showing increased phage diversity relative to their household controls. These virome changes in disease states were inversely proportional to bacterial diversity and richness. The authors suggest these observations demonstrate an expansion of lytic phages, possibly by prophage induction. This work opened the question on the role phages have in contributing to the pathogenesis in IBD. The same dataset used in the study by Norman and colleagues was later re-analysed at a lower taxonomic resolution to overcome inter-individual variation, with contrasting results, highlighting the importance of standardising bioinformatics pipelines for viral read analyses to prevent bioinformatics artefactual data (Clooney et al., 2019). This study did not find any differences in viral richness between IBD patients and healthy controls, although they did see an increased diversity of *Caudovirales* within CD patients. They also show there was an increase of temperate viral clusters in patients with CD compared to controls and suggest that a switch to a lysogenic phage life cycle is associated with disease, while the healthy core virome is predominantly composed of lytic phages (Clooney et al., 2019). The authors suggest that environmental stresses can easily trigger prophage induction due to the high abundance of prophages in this disease state, thereby worsening inflammation by the release of antigens and pathogen-associated molecular patterns upon bacterial host lysis, that consequently trigger inflammation cascades.

Moreover, there have been suggestions that the presence or absence of specific members of the microbiome are associated with IBD. For instance, Guerin and colleagues identified that crAss-like phage counts were lowest in IBD patients (United States cohort) although no disease association analyses were conducted (Guerin et al., 2018). Contrastingly, in the study by Norman and colleagues, the presence of the crAssphage was found in 71% of the samples tested with as much as 89% of the reads belonging to it (Norman et al., 2015). However, its presence did not correlate with disease status (Norman et al., 2015; Edwards et al., 2019). Notably, they only searched for the prototype crAssphage and thus could have missed associations with other crAss-like phages. crAss-like phages have been established as part of the core human gut phageome (Manrique et al., 2016) and therefore whether these particular phages – or others – have implications in homeostasis and/or disease states needs to be further investigated.

Work by Federici and colleagues demonstrated an enriched clade of *Klebsiella pneumoniae* (ST323) to be significantly associated with IBD severity and gut inflammation on a global scale. The authors additionally explored phage therapy as a potential therapeutic, with decreases in *K. pneumoniae* load and in pro-inflammatory cytokines (Federici et al., 2022a,b). The mechanisms underpinning these phage-mediated benefits remain unknown.

In order to speculate how phages may be modulating human health, we must first understand how phages exist within the mucus layer. Phage residence in the mucus layer is not only attributed to mucus-binding proteins, but also to phage particle charge, mucus secretion and turnover dynamics, and mucus properties including pore size (~100–500 nm (Round et al., 2012; Witten and Ribbeck, 2017)), structure, and viscosity (Barr et al., 2015; Joiner et al., 2019; Kaler et al., 2022). All these factors will influence phage diffusion and transit through the mucus layer.

Three diffusion modes are considered to be relevant in mucosal structures: Brownian motion (BM), fractional Brownian motion (FBM) and continuous time random walk (CTRW; Kaler et al., 2022). Phage particle movement in mucosal surfaces has only been studied on phage T4 (~200 nm length) in commercially available mucin (Barr et al., 2015; Joiner et al., 2019). In these studies, the phage particle diffusion mode changed depending on the mucin concentration, with sub-diffusion (CTRW) being predominant at ≥ 1% w/v mucin concentrations. This led to the hypothesis that phages in the gut will first encounter the top mucus layer (low mucin concentration), and those that can bind mucus will transiently bind and initiate sub-diffusion down the mucus, closer to the epithelia, where phages may temporarily be ‘stuck’ as mucin concentration is higher (Figure 3). This suggests higher phage density closer to the epithelia, due to a reduced transit speed where phage clearance is dependent on continuous mucus secretion and turnover (Barr et al., 2015).

A separate study tracked nanoparticle (~100 nm diameter) movement through human airway mucus and found that it could diffuse by all three diffusion modes, with FBM predominating (Kaler et al., 2022). BM particles diffuse more effectively, followed by CTRW, with FBM being the least effective. In line with previous T4 studies, Kaler and colleagues show that mucin density affected the proportion of the diffusion modes seen, as well as the speed of mucosal crossing for all particles. Thus, more particles diffused by FBM as confinement increased, meaning particle movement was slower in highly dense mucin. Some insight can be gained from studies on eukaryotic viruses, given that some respiratory viruses require the efficient penetration of the mucus layer to infect the mammalian cells. For instance, Kaler and colleagues additionally tested diffusion of the influenza A virus (~100 nm) through the mucus layer and found that only the few particles that diffused by BM were able to cross the barrier. The authors suggest that the hemagglutinin and neuraminidase proteins on the virus envelope interact with mucins in such a way that it allows viral particle movement through the mucus (e.g., hemagglutinin binds to sialylated mucins, and neuraminidase cleaves the glycans). Steric and adherence interactions were attributed to restricting the movement of the other particles that were trapped (Kaler et al., 2022). We suspect phages in the gut may have similar mucin-adherence and catalytic interactions as this influenza A viral particle. It will be interesting to compare these results to different phage particles and determine if the previous findings on phage T4 extend to endogenous mucin structures (including mucins from the small versus large intestine).

Thus, in the context of disease, it is possible that the microbial dysbiosis (bacteriome and virome) seen in gastrointestinal diseases that manifest mucosal dysfunction, like IBD, is associated with defective phage-mucus dynamics. For instance, the lack of mucus secretion or faulty mucin glycosylation could lead to a more concentrated pool of phages closer to the epithelia that may trigger inflammation responses, due to decreased mucus turnover and different binding sites. A concentrated pool of phages could also increase prey-encounter rates, leading to decreases in commensal bacterial populations, allowing the colonisation and persistence of pathogenic bacteria (e.g., *K. pneumoniae* ST323). Environmental stress could additionally be increasing the phage population by prophage excision from the host. Equally, in healthy states, we hypothesise different phages have different motility rates through the mucus depending on their size, charge and encoded proteins. For instance, phage-encoded proteins presenting higher affinity to mucins might persist for longer in the mucosal layer. Similarly, phage proteins with both binding and catalytic interactions (like the Influenza A virus) may further influence phage persistence in the mucus.

Overall, it becomes apparent that there exists a large gap in the field that needs to be explored to increase our understanding of phage-mediated effects in the gut. Studies investigating phage populations in the gut are a starting point to address this knowledge gap (Dutilh et al., 2014; Devoto et al., 2019; Benler et al., 2021; Camarillo-Guerrero et al., 2021; Yutin et al., 2022). These studies should be complemented with an increased characterisation of gut phage populations, including functional analysis of their encoded proteins and particularly of those that may mediate mucosal interactions. We should also consider phage diffusion rates across different types of endogenous mucin structures, the mechanisms underpinning these (e.g., enzymatic functions and/or affinities), and how these factors change in disease states.

## Conclusion

The understanding of phage-mucus interacting proteins in human health modulation is lacking and challenged by the difficulty of characterising gut phages and testing in *in vivo* models. Major efforts are needed to advance our knowledge in this area to levels comparable or superior to what is currently known about bacteria-mucus-human dynamics. Studies aiming at determining spatial distribution, phage protein activity, and phage-mediated modulation of human health within the human gastrointestinal tract, will all increase our understanding of phages in the gut.

In this review, we have discussed the importance of mucus mucins and of bacteria- and phage-mucus interactions. We discussed the known and suggested non-gut-specific phages that encode proteins or domains with mucin-interacting activities. We hypothesise that mucosal-adhering phages in the mammalian gut will have evolved, like bacteria, to encode such proteins, allowing their persistence in the mucosal layer and interaction with their hosts. Additionally, we propose that phages, *via* mucus interactions, could have direct and/or indirect effects on the human ‘host’, using IBD (a mucosal-associated disease) as an example.

To demonstrate phage-mucus interactions and their role in modulating human health, we first need to characterise the proteome of known gut phages and identify more phage-encoded proteins with mucus-interacting (both binding and degrading) functions. To do so, we suggest that structural protein comparisons and computational methods may offer powerful ways to identify relevant proteins, which will require subsequent experimental validation. For mucus-adherent phages, it will be relevant to study the dynamics that allow their persistence including diffusion rates, and how they change in disease states. A collaborative effort is needed to explore this field, and allow us to deepen the knowledge of phage-mediated interactions in the mammalian gut.

## Author contributions

DR-R wrote the manuscript. MH illustrated the figures. MK and SK reviewed the manuscript. FN helped in the envisioning of the manuscript and reviewed all drafts of the paper. All authors contributed to the article and approved the submitted version.

## Funding

This work was funded by Bowel Research UK.

## Conflict of interest

The authors declare that the research was conducted in the absence of any commercial or financial relationships that could be construed as a potential conflict of interest.

## Publisher’s note

All claims expressed in this article are solely those of the authors and do not necessarily represent those of their affiliated organizations, or those of the publisher, the editors and the reviewers. Any product that may be evaluated in this article, or claim that may be made by its manufacturer, is not guaranteed or endorsed by the publisher.
